# Cerebellar contribution to emotional body language perception: a TMS study

**DOI:** 10.1093/scan/nsz074

**Published:** 2019-10-07

**Authors:** Chiara Ferrari, Andrea Ciricugno, Cosimo Urgesi, Zaira Cattaneo

**Affiliations:** Department of Psychology, University of Milano–Bicocca, Milan 20126, Italy; Department of Brain and Behavioral Sciences, University of Pavia, Pavia 27100, Italy; IRCCS Mondino Foundation, Pavia 27100, Italy; Laboratory of Cognitive Neuroscience, Department of Languages and Literatures, Communication, Education and Society University of Udine, Udine 33100, Italy; Scientific Institute, IRCCS E. Medea, Neuropsychiatry and Neurorehabilitation Unit, Bosisio Parini, Lecco 23900, Italy; Department of Psychology, University of Milano–Bicocca, Milan 20126, Italy; IRCCS Mondino Foundation, Pavia 27100, Italy

**Keywords:** cerebellum, emotions, TMS, body expressions

## Abstract

Consistent evidence suggests that the cerebellum contributes to the processing of emotional facial expressions. However, it is not yet known whether the cerebellum is recruited when emotions are expressed by body postures or movements, or whether it is recruited differently for positive and negative emotions. In this study, we asked healthy participants to discriminate between body postures (with masked face) expressing emotions of opposite valence (happiness *vs* anger, Experiment 1), or of the same valence (negative: anger *vs* sadness; positive: happiness *vs* surprise, Experiment 2). While performing the task, participants received online transcranial magnetic stimulation (TMS) over a region of the posterior left cerebellum and over two control sites (early visual cortex and vertex). We found that TMS over the cerebellum affected participants’ ability to discriminate emotional body postures, but only when one of the emotions was negatively valenced (i.e. anger). These findings suggest that the cerebellar region we stimulated is involved in processing the emotional content conveyed by body postures and gestures. Our findings complement prior evidence on the role of the cerebellum in emotional face processing and have important implications from a clinical perspective, where non-invasive cerebellar stimulation is a promising tool for the treatment of motor, cognitive and affective deficits.

## Introduction

The cerebellum is known to play an important role in emotion regulation, having recently been recognized as an integral part of the limbic network that underpins affective processing (Adamaszek *et al.*, 2017; Schmahmann, 2019) and as a key node of the ‘social brain’ (Van Overwalle *et al.*, 2014, 2019). Specifically, neuroimaging findings consistently point toward the posterior vermis and to Crus I and Crus II (in the cerebellar hemispheres) as regions involved in mediating the perception of others' emotional states (for meta-analyses see Stoodley and Schmahmann, 2010; Keren-Happuch *et al.*, 2014; see also Guell *et al.*, 2018). Accordingly, cerebellar lesions can affect the recognition of basic facial and social emotions (Adamaszek *et al.*, 2015; Clausi *et al.*, 2018; D'Agata *et al.*, 2011; Hoche *et al.*, 2016). Cerebellar dysfunctions have also been reported in patients with psychiatric syndromes, such as schizophrenia and autism, which are characterized by emotional deficits (Mothersill *et al.*, 2016; Sathyanesan *et al.*, 2019). Brain stimulation experiments further support this pattern: interfering with cerebellar activity by non-invasive brain stimulation affects emotional processing in healthy participants, even when the emotional cues are task-irrelevant (Ferrari *et al.*, 2018a; Ferrucci *et al.*, 2012; Schutter *et al.*, 2009). Furthermore, delivering transcranial magnetic stimulation (TMS) over the cerebellum has been found to modulate theta and gamma frontal activity (Schutter *et al.*, 2003; Schutter and van Honk, 2006), which are neural oscillations that are related to emotional processing (for a review, Symons *et al.*, 2016).

Prior studies investigating the cerebellar contribution to the processing of emotions that are expressed by other agents have mostly employed faces as stimuli (Ferrari *et al.*, 2018a; Ferrucci *et al.* 2012; Schutter and van Honk, 2006; Schraa-Tam *et al.*, 2012). However, the body is also critical in conveying information about an individual’s emotional state (de Gelder *et al.*, 2015). Indeed, emotions can be accurately recognized by looking at bodily postures and movements in the absence of facial information, even at very short presentation times (e.g. 250 ms, Martinez *et al.*, 2016; see also Atkinson *et al.*, 2004; Ferrari *et al.*, 2019). Although no functional magnetic resonance imaging (fMRI) study has specifically investigated cerebellar responses during recognition of body emotions, cerebellar activation has been observed when viewing hand gestures that are symbolic of different valences (e.g. ‘thumbs up’, Lindenberg *et al.*, 2012; Prochnow *et al.*, 2013), as well as when viewing dynamic emotional bodies (Peelen *et al.*, 2007). Moreover, when judging emotions expressed by stick figure characters (with no facial information available), self-reported empathizing was found to significantly predict activation in bilateral cerebellar sectors (Kana and Travers, 2012; see also Jastorff *et al.*, 2015). Despite this neuroimaging evidence, the ‘causal’ contribution of different cerebellar sectors in mediating the processing of bodily emotions remains to be investigated.

It is also not clear whether the cerebellar involvement in emotional processing is valence-specific. Although the cerebellum appears to be involved in the processing of both positive and negative emotions (Baumann and Mattingley, 2012; D'Agata *et al.*, 2011; Sato *et al.*, 2019), converging evidence from neuroimaging, brain stimulation and patient studies suggests that the cerebellum may be more robustly engaged by negatively valenced stimuli (Adamaszek *et al.*, 2014; Ferrucci *et al.*, 2012; Park *et al.*, 2010; Schraa-Tam *et al.*, 2012; for a review see Leggio and Olivito, 2018). For instance, while the perception of positive emotional faces evoked only mild cerebellar activations, negative emotional faces evoked prominent activations in several cerebellar structures (Schraa-Tam *et al.*, 2012). In line with this, the fMRI study of Park *et al.* (2010) revealed activations in the cerebellum exclusively for anger but not for happiness. Moreover, applying transcranial direct current stimulation over the cerebellum significantly enhanced sensory processing in response to negative facial expressions, leaving positive and neutral facial expressions unchanged (Ferrucci *et al.*, 2012). In fact, when compared to positive stimuli, negatively valenced stimuli may prompt a goal-directed behavior (for which the cerebellum is relevant, see Manto *et al.*, 2012) to react to another agent’s (negative) expressions (Schraa-Tam *et al.*, 2012). However, the opposite pattern of activation has also been reported (Hoche *et al.*, 2016; Peelen *et al.*, 2007; Schutter *et al.*, 2009). In particular, Schutter *et al.* (2009) found that modulating activity in the vermis with TMS affected implicit processing of facial expressions of happiness but not of fear. Similarly, in a neuroimaging study in which participants were asked to indicate how much a body (with masked face) matched a specific emotion provided by the experimenter, Peelen *et al.* (2007) found selective cerebellar involvement for positive emotions. Moreover, patients with cerebellar disorders were more impaired in recognized positive emotions than negative ones when only the eye region of the face was shown (Hoche *et al.*, 2016). Therefore, it is not clear whether the valence of the emotional expression is relevant to the activation of the cerebellum. Finally, prior findings suggest that emotion recognition and neural responses in the emotional brain might be affected by the perspective (facing the observer or averted) in which faces (e.g. Sauer *et al.*, 2013) and bodies (Soria Bauser *et al.*, 2012) are presented. The ‘facing’ orientation prompts the impression of being involved in a dyadic interpersonal interaction (Soria Bauser *et al.*, 2012), and may thus activate the cerebellum more, possibly by triggering motor preparation or emotional resonance, more so than bodies that are oriented away from the observer.

In the current study, we employed TMS to shed light on the contribution of the cerebellum to the processing of emotional body expressions. In two experiments, we asked participants to discriminate between emotions expressed by body postures of different valence (anger *vs* happiness) or of the same valence (i.e. negative: anger *vs* sadness and positive: happiness *vs* surprise). We expected that TMS over the cerebellum would interfere with participants’ ability to discriminate between the valence of emotional body expressions, compared to stimulation over the control sites. This hypothesized pattern of results would be consistent with the effect of cerebellar TMS on facial emotion discrimination (Ferrari *et al.*, 2018a), and in line with neuroimaging evidence showing cerebellar responses to body emotional expressions (Lindenberg *et al.*, 2012; Peelen *et al.*, 2007; Prochnow *et al.*, 2013). Moreover, if cerebellar recruitment during emotional processing is particularly critical for negative emotions (Ferrucci *et al.*, 2012; Park *et al.*, 2010; Schraa-Tam *et al.*, 2012), cerebellar TMS may affect the discrimination of negative expressions more than positive emotions (but see Hoche *et al.*, 2016; Peelen *et al.*, 2007; Schutter and van Honk, 2009). Finally, if greater activation occurs for directly oriented stimuli, cerebellar TMS may have a greater effect on the processing of emotions expressed by bodies oriented toward the observer, compared to those that are averted away.

## Experiment 1

In Experiment 1, we aimed to test the contribution of the cerebellum in processing emotional body expressions, by asking participants to discriminate between static angry and happy body postures, while receiving TMS over the cerebellum or over two control sites. For cerebellar stimulation, we selectively targeted a region of the posterior (medial) left cerebellum, at the boundaries of Crus I/Crus II and vermal lobule VII. Although emotional processing drives bilateral activation in vermis lobules VI–VII and Crus I/II (Keren-Happuch *et al.*, 2014; Guell *et al.*, 2018; Stoodley and Schmahmann, 2009), recent findings indicate that there may be a greater engagement of the left posterior cerebellum in response to emotional stimuli (Sato *et al.*, 2019). Therefore, we targeted a left cerebellar site. In line with this, Cho *et al.* (2012) showed that delivering inhibitory repetitive TMS over the left lateral cerebellum resulted in a decrement of glucose metabolism in orbitofrontal, medial frontal and anterior cingulate gyri, regions typically related to emotional processing (see also Halko *et al.*, 2014).

**Figure 1:**
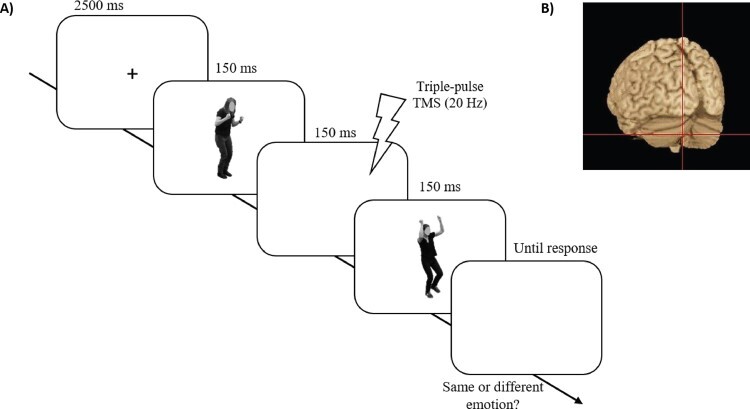
(**A**) Timeline of an experimental trial in Experiment 1. Each trial started with a fixation cross (2500 ms), followed by the first body (150 ms), a blank screen (150 ms) and then by the second body (150 ms). Participants had to indicate whether the two bodies expressed the same or different emotions (in the example shown, the first body is expressing anger and the second body happiness). Triple-pulse TMS (20 Hz) was delivered between the offset of the first body and the onset of the second body. (**B**) Targeted cerebellar loci of stimulation: left Crus I/II (*x* = −9, *y* = −76, *z* = −32, TAL) as shown in MRIcro template.

## Methods

### Participants

Twenty volunteers took part in the study (11 males, mean age = 23.2 years, SD = 1.6). All participants were right-handed and had normal or corrected-to-normal vision. Prior to the TMS experiment, each participant filled in a questionnaire to evaluate compatibility with TMS (translated from Rossi *et al.*, 2011). The protocol was approved by the local ethics committee and participants were treated in accordance with the Declaration of Helsinki.

### Stimuli

Stimuli were images selected from the Bochum Emotional Stimulus Set (Thoma *et al.*, 2013) depicting eight different male and eight different female young-adult bodies (with masked faces). Each body (covering ~23 × 14 degrees of visual angle) was presented four times; once in each of the four possible combinations of expressed emotion (happiness *vs* anger) and orientation (facing the observer *vs* averted by 45° to their left). We selected the bodies so that the emotion they expressed was highly recognizable, with accuracy recognition rates exceeding 80% (as reported in the validation study, Thoma *et al.*, 2013). The frontal and averted bodies that were selected from the original database did not differ in the recognizability of their emotional expressions (pairwise comparison for recognition rates of selected frontal *vs* averted bodies: *P* = 0.28 for angry bodies and *P* = 0.18 for happy bodies); this ensures that any difference due to TMS is not the result of a general trend for poorer recognition of averted body expressions.

### Procedure

Participants were seated in front of a 19° screen at an approximate distance of 57 cm. The software E-prime 2.0 (Psychology Software Tools, Pittsburgh, PA) was used for stimulus presentation, data collection and TMS triggering. Figure 1 shows an example of an experimental trial. Each trial started with a black fixation cross appearing in the middle of the screen (2500 ms), followed by the first body (visible for 150 ms), a blank screen (150 ms) and then a second body (150 ms). The second body was followed by the presentation of a blank screen, during which time participants’ responses were recorded (responses given before the offset of the second body stimulus were not recorded). Participants were required to indicate whether the two presented bodies expressed the same or different emotions (without having to name the specific emotion). Participants responded as quickly as possible by using their right hand to press the left or right arrow key. Response key assignment was counterbalanced across participants. After a short training session consisting of eight trials, participants performed three experimental blocks, one for each TMS site (see ‘TMS’ below). The order of site stimulation was counterbalanced across participants. Each block consisted of 64 trials repeated twice, for a total of 128 trials in each block. Half of the trials featured body pairs expressing the same emotion (happy–happy or angry–angry) and the other half expressing a different emotion (happy–angry or angry–happy). Same and different trials were intermixed within each block. Moreover, half of the trials consisted of images depicting body pairs from a frontal view and the other half consisted of images of body pairs depicted from an averted view. Within each trial, the bodies were always depicted from the same view (either frontal or averted) and were always of the same gender (but of different identities).

### TMS

Online neuronavigated TMS was performed with a Magstim Rapid^2^ stimulator (Magstim Co, Ltd, Whitland, UK) connected to a 70-mm butterfly coil. At the beginning of each session, single-pulse TMS was applied over the left M1 at increasing intensities to determine each participant’s resting motor threshold (rMT). rMT was defined as the minimal intensity of the stimulator output that produced motor evoked potentials (the motor response measured through electrodes applied to the hand muscles) with amplitude of at least 50 mV in the first dorsal interosseous with 50% probability (Rossini *et al.*, 1994; see also Hanajima *et al.*, 2007 for methodological details on this standard procedure). Participants were stimulated at 100% of their rMT, which is consistent with prior TMS studies targeting the cerebellum (e.g. Demirtas-Tatlidede *et al.*, 2011; Ferrari *et al.*, 2018b). The intensity of stimulation was kept constant for the stimulation of all three target sites and corresponded to 48.5% of the maximum stimulator output (SD = 3.5). Triple-pulse 20-Hz TMS was delivered in each experimental trial 150 ms before the presentation of the second body. 20-Hz rTMS effectively modulated behavioral responses in previous TMS studies, also targeting the cerebellum (e.g. Bestmann *et al.*, 2002; Cattaneo *et al.*, 2014a; Gamond *et al.*, 2017; Koch *et al.*, 2007; Wagner *et al.*, 2006). TMS was delivered over a region in the left cerebellum, while the early visual cortex and the vertex were targeted as control sites. The early visual cortex was chosen as an additional control area beyond the vertex since prior evidence suggests that cerebellar stimulation may spread to the primary visual cortex (Renzi *et al.*, 2014); therefore, it is important to rule out the possibility that the effect of cerebellar TMS is due to the indirect stimulation of the visual cortex. The cerebellar target region and the early visual cortex were localized by means of stereotaxic navigation on individual estimated magnetic resonance images (MRIs) obtained through a 3D warping procedure fitting a high-resolution MRI template with the participant's scalp model and craniometric points (Softaxic 3.0, EMS, obtained using individual MRI scans, see Carducci and Brusco, 2012). This localization procedure has been successfully used in many prior TMS studies (e.g. Balconi and Ferrari, 2013; Cattaneo *et al.*, 2011, 2014b, 2015; Ferrari *et al.*, 2016). The anatomical Talairach coordinates (Talairach and Tournoux, 1998) of the cerebellum (Tal *x* = −9, *y* = −76, *z* = −32) were taken from a prior neuroimaging study reporting activation in this cerebellar sector during processing of bodies (Grezes *et al.*, 2007) and corresponded to a region of the posterior medial left cerebellum that has been found to be active during emotional processing (Guell *et al.*, 2018). For localization of the early visual cortex, we referred to prior neuroimaging evidence (Tal *x* = −2, *y* = −75, *z* = 32, Anderson *et al.*, 2011). The vertex was localized as the point falling half the distance between the nasion and the inion on the same midline. For the vertex stimulation, the coil was placed tangentially to the scalp and held parallel to the midsagittal line with the handle pointing backward. For cerebellar and early visual cortex stimulation, the coil was placed tangentially to the scalp and held parallel to the midsagittal line with the handle pointing superiorly (see Figure 1B), which is consistent with evidence suggesting that this is an effective coil orientation to successfully modulate activity in cerebellar structures (e.g. Bijsterbosch *et al.*, 2012; van Dun *et al.*, 2017). No participant reported phosphenes during the experiment.

## Statistical analysis and results

Mean accuracy rates and mean reaction times (RTs, recorded from the offset of the second stimulus) were computed for each participant in each experimental condition. Response latencies that were +/−3 SD compared to each participant’s block mean were excluded from the analyses (following this criterion, 2.0% of total trials were excluded). Accuracy scores and RT for correct responses were analyzed using separate repeated-measures ANOVAs, with body view (frontal *vs* averted) and TMS site (left cerebellum, early visual cortex and vertex) as within-subjects variables.

**Figure 2:**
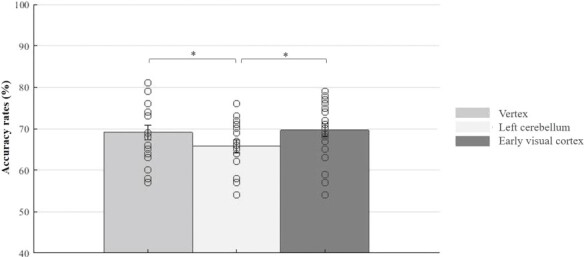
Accuracy rates (%) as a function of TMS site (vertex, left cerebellum and early visual cortex) in Experiment 1 (data collapsed across body orientation). TMS over the left cerebellum significantly impaired participants’ accuracy in discriminating between happy and angry bodies compared to TMS over the vertex and the early visual cortex. Error bars indicate ±1 SEM and data points represent individual values. Asterisks indicate a significant difference (*P* < 0.05, Bonferroni corrected) across TMS conditions.

The ANOVA on mean accuracy scores revealed a significant main effect of TMS site, *F*(2,38) = 6.52, *P* = 0.004, indicating that TMS over the left cerebellum lowered participants ability to discriminate between emotional body postures compared to TMS over the early visual cortex, *t*(19) = 3.13, *P* = 0.017, and the vertex, *t*(19) = 3.80, *P* = 0.004 (Bonferroni corrections applied, see Figure 2). No difference in accuracy was observed between stimulation over the early visual cortex and stimulation of the vertex, *t*(19) < 1, *P* = 1.00 (Bonferroni correction applied). Neither the main effect of body view, *F*(1,19) < 1, *P* = 0.46, nor the interaction between body view and TMS site, *F*(2,38) < 1, *P* = 0.95, reached significance.

Mean RT for correct responses was 787 ms (SD = 266) for the left cerebellar stimulation, 793 ms (SD = 306) for the early visual cortex stimulation and 821 ms (SD = 331) for the vertex stimulation. The ANOVA on mean RT revealed no significant main effect of TMS site, *F*(2,38) < 1, *P* = 0.84, no significant main effect of body view, *F*(1,19) < 1, *P* = 0.21 and no significant interaction between TMS site and body view, *F*(2,38) < 1, *P* = 0.86.

## Experiment 2

In Experiment 1, we found that applying TMS over a region of the posterior medial left cerebellum affected participants’ ability to discriminate between body emotional expressions of happiness and anger, suggesting that this region is critically involved in perceiving others’ emotional states when conveyed by body postures. However, the data of Experiment 1 do not allow us to disentangle any valence-specific effects of cerebellar stimulation. To address this aim, in Experiment 2 we asked participants to discriminate either between two negative emotions (anger and sadness) or between two positive emotions (happiness and surprise). Moreover, although in Experiment 1 we found no effect of body orientation, in Experiment 2 we kept this manipulation to enable us to make a more direct comparison between the two experiments (while also presenting participants with more variation in the stimuli).

## Methods

### Participants

Forty Italian volunteers (13 males, mean age = 24.0 years, SD = 4.6) took part in the experiment. All participants were right-handed, had normal or corrected-to-normal vision and had not participated in Experiment 1. Inclusion criteria were the same as for Experiment 1. The protocol was approved by the local ethics committee and participants were treated in accordance with the Declaration of Helsinki.

### Stimuli, procedure and TMS

The procedure was similar to Experiment 1, but this time participants were randomly assigned to discriminate between either anger and sadness (negative emotions condition) or between happiness and surprise (positive emotions condition) in a between-subjects design. A between-subjects design was necessary to keep a sufficiently high number of trials (ensuring appropriate statistical power) while keeping the number of pulses delivered to each participant within TMS safety limits (Rossi *et al.*, 2009). Bodies expressing anger and happiness were the same as those used in Experiment 1; bodies expressing sadness and surprise were taken from the same database (Thoma *et al.*, 2013). We selected sad and surprised bodies whose recognizability rates were >80% as reported in the validation study (Thoma *et al.*, 2013). Importantly, recognizability of sadness and surprised emotions did not vary as a function of body orientation (comparison between recognition rates of frontal *vs* averted bodies: *P* = 0.36 for sad bodies and *P* = 0.11 for surprised bodies). As in Experiment 1, each block consisted of 64 trials, repeated twice (for a total of 128 trials in each block), half of which represented body pairs expressing the same emotional valence (either happiness or surprise in one group, and either sadness or anger in the other group) and the other half expressing a different emotional valence (happiness *vs* surprise in one group, and anger *vs* sadness in the other group). As in Experiment 1, half of the trials consisted of images depicting body pairs from a frontal view and the other half consisted of images of body pairs depicted from an averted view. Within each trial, the bodies were always depicted from the same view (either frontal or averted) and were always of the same gender. TMS parameters were the same as those used in Experiment 1. Mean stimulation intensity was set for each participant as in the previous experiment and corresponded to 50.4% of the maximum stimulator output (SD = 2.9). No participant reported phosphenes during the experiment.

## Statistical analysis and results

As in Experiment 1, trials in which participants' RTs (recorded from the offset of the second body stimulus) were +/−3 SD compared to their block mean were excluded from the analyses (following this criterion, 1.4% of total trials were excluded). Mean accuracy rates and RT for correct responses were analyzed using separate repeated-measures ANOVAs with body view (frontal *vs* averted) and TMS site (left cerebellum, early visual cortex and vertex) as within-subjects variables and emotional valence condition (positive emotions, negative emotions) as a between-subjects factor.

**Figure 3:**
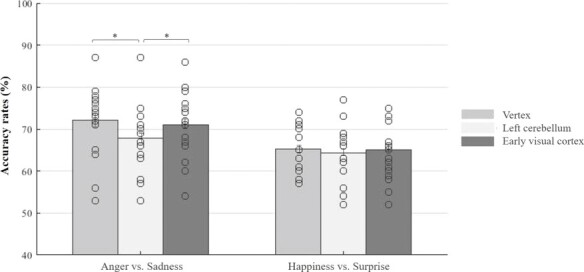
Accuracy rates (%) as a function of TMS site (vertex, left cerebellum and early visual cortex) and valence of the emotion conveyed by the bodies in Experiment 2 (data collapsed across body orientation). Left cerebellar TMS significantly lowered participants’ ability to discriminate between negative emotions of anger and sadness compared to TMS over the vertex and the early visual cortex. In turn, left cerebellar TMS did not affect participants’ accuracy in discriminating between positive emotions of happiness and surprise. Error bars indicate ±1 SEM and data points represent individual values. Asterisks indicate a significant difference (*P* < 0.05, Bonferroni corrected) across TMS conditions.

The ANOVA on mean accuracy scores revealed significant main effects of body view, *F*(1,38) = 7.16, *P* = 0.011, and emotional valence, *F*(1,38) = 6.96, *P* = 0.012, as well as a significant interaction effect between body view and emotional valence, *F*(1,38) = 12.49, *P* = 0.001. The main effect of TMS site, *F*(2,76) = 8.46, *P* < 0.001, and the interaction between TMS site and emotional valence, *F*(2,76) = 3.16, *P* = 0.048, also reached significance. None of the other interaction effects was significant (all *P*s > 0.21). The significant main effects of body view and emotional valence were analyzed in light of their significant interaction. Participants presented with the negative valence condition were better at discriminating between angry and sad bodies in the averted (mean accuracy: 73%, SD = 7.8) than in the facing orientation (mean accuracy: 69%, SD = 7.7) condition, *t*(19) = 3.69, *P* = 0.002. In turn, participants presented with happy and surprised bodies discriminated between them with a similar level of accuracy in the averted (mean accuracy: 65%, SD = 6.8) and facing orientations (65%, SD = 5.3), *t*(19) < 1, *P* = 0.73. To clarify the significant TMS site by emotional valence interaction, we conducted an analysis of the main effect of TMS within each emotional valence condition. For the participants that were presented with happy and surprised bodies, the main effect of TMS did not reach significance, *F*(2,38) < 1, *P* = 0.48. Conversely, for the group presented with angry and sad bodies, the main effect of TMS was significant, *F*(2,38) = 9.62, *P* < 0.001. *Post-hoc* comparisons showed that left cerebellar TMS impaired discrimination of sad and angry bodies compared to TMS over the early visual cortex, *t*(19) = 2.77, *P* = 0.037, and the vertex, *t*(19) = 5.06, *P* < 0.001 (Bonferroni corrections applied, see Figure 3). No difference in accuracy was observed for TMS over the early visual cortex and the vertex, *t*(19) = 1.04, *P* = 0.93 (Bonferroni correction applied).

Mean RTs for correct responses were 590 ms (SD = 203) for the left cerebellar stimulation, 599 ms (SD = 164) for the vertex stimulation and 592 ms (SD = 169) for the early visual cortex stimulation. The ANOVA on mean RT for correct responses revealed non-significant main effects of body view, *F*(1,38) = 1.05, *P* = 0.31, and TMS, *F*(2,76) < 1, *P* = 0.91. The interaction between body view and emotional valence reached significance, *F*(1,19) = 14.38, *P* = 0.001. Pairwise comparisons indicated that participants were faster to respond when discriminating between sad and angry bodies in the averted (mean RT = 582 ms, SD = 139) than in the frontal view condition (mean RT = 603 ms, SD = 159), *t*(19) = 2.37, *P* = 0.027. Body orientation did not affect RT for participants discriminating between surprised and happy bodies, *t*(19) = 1.22, *P* = 0.24 (mean RT for averted bodies = 576 ms, SD = 182; mean RT for frontal bodies = 562 ms, SD = 177).

## Discussion

In two experiments, we found that interfering with cerebellar activity via online TMS affected participants’ ability to discriminate between anger and other emotions of same (i.e., sadness) or opposite (i.e., happiness) valence, but not with the ability to discriminate between two positively valenced emotions (surprise *vs* happiness). Overall, our findings suggest that the left medial posterior cerebellum is crucially implicated in coding the emotional content of bodies showing negative emotions. Although it has previously been suggested that the cerebellum is a node of the emotional brain that supports understanding of others’ emotions (for a review see Adamaszek *et al.*, 2017), our study is the first to demonstrate a specific ‘causal’ contribution of this structure in processing emotional body gestures and postures. Our findings fit well with previous neuroimaging investigations that observed posterior cerebellar activations in response to emotional body postures and gestures (e.g. Jastorff *et al.*, 2015; Kana and Travers, 2012; Peelen *et al.*, 2007) and with the results of a recent analysis on a large fMRI database indicating that left-posterior sectors of the cerebellum (left Crus II and left lobule VI, as well as Crus II vermis) may be critically involved in processing emotions (Guell *et al.*, 2018; see also Sato *et al.*, 2019).

Interestingly, our study also revealed valence-specific effects, such that cerebellar TMS affected emotion discrimination only in trials in which at least one negative emotion was displayed (anger *vs* happiness in Experiment 1 and anger *vs* sadness in Experiment 2). This finding is consistent with previous neuroimaging and neurostimulation studies that reported preferential involvement of the cerebellum when processing negative emotional stimuli (Ferrucci *et al.*, 2012; Park *et al.*, 2010; Schraa-Tam *et al.*, 2012; for a review see Leggio and Olivito, 2018). However, our findings are inconsistent with the fMRI study by Peelen *et al.* (2007) that showed selective activation of a left-lateralized cerebellar cluster in response to happy bodies but not to bodies expressing the basic negative emotions (but that the region activated in Peelen *et al.*, 2007, was more anterior than the region we targeted). Whether representations of different emotional valence are hosted by different cerebellar subregions (also depending on the task at play) is an interesting open question. In this regard, the selective effect of left-cerebellum TMS on the discrimination of negative emotions may be interpreted as providing support for the notion that valence-specific responses are lateralized in the cerebellum, as it has been observed in the cerebrum (e.g. Adolphs *et al.*, 2001; but see Ferrari *et al.*, 2017; Lindquist *et al.*, 2015). Indeed, networks that are strongly lateralized within the cerebrum seem also to be strongly lateralized within the cerebellum (Wang *et al.*, 2012). However, we do not have data from right cerebellar TMS to support this hypothesis. In light of consistent evidence showing that vermal as well as right-lateral sectors of the posterior cerebellum are involved in emotional processing (and social cognition tasks) (see meta-analyses by Keren-Happuch *et al.*, 2014; Stoodley and Schmahmann, 2009; Van Overwalle *et al.*, 2014), TMS over these regions may also affect facial and body emotions recognition, a possibility that needs empirical testing.

The mechanisms through which the cerebellum contributes to emotional processing remain to be clarified. One possibility is that the cerebellum takes part in processing body emotional expressions by implementing mirror-based mechanisms. Indeed, the cerebellum has been found to several cerebellar regions known to specifically respond to the observation and imitation of emotional expressions (Leslie *et al.*, 2004; Schraa-Tam *et al.*, 2012; for mirror properties of the cerebellum see also the meta-analysis by Molenberghs *et al.*, 2012). Moreover, Likowski *et al.* (2012) showed that activity in the lateral cerebellum correlated with activity in facial muscles (assessed by electromyography) in response to the observation of others' emotional facial expressions. However, the cerebellar sectors involved in aspects of somatosensory and motor control (Schmahmann, 2010; Stoodley and Schmahmann, 2010) are located in the anterior part of the cerebellum, which is unlikely to be effectively reached by TMS (at least, when using a standard figure of eight 70-mm coil, as we did, see Fernandez *et al.*, 2018; Hardwick *et al.*, 2014). We specifically targeted a region (Crus I/II) of the posterior (left) cerebellum that, due to its anatomical and functional connections to the associative cortical regions (Kelly and Strick, 2003), is likely to be involved in the more reflective, cognitive components of affective and social tasks (Adamaszek *et al.*, 2017; Sokolov *et al.*, 2017). It has been proposed that the (posterior) cerebellum participates in higher-order functions by generating simulations of events (i.e. motor or mental actions) in the form of internal models (e.g. Courchesne and Allen, 1997; Ito, 2008; Leggio and Molinari, 2015; Manto *et al.*, 2012; Peterburs *et al.*, 2015; Schmahmann, 2010; for reviews see Bellebaum *et al.*, 2012; D'Angelo and Casali, 2013). As noted by Sokolov *et al.* (2017), having a sense of other individuals’ emotional state requires not only the creation of a mental model of those individuals but also the capacity to simulate how these mental states might influence their behavior. It may be that the cerebellar TMS in our study affected these simulation-like mechanisms, which made emotional discrimination harder in our task, at least when the emotions were evolutionary salient (as it is the case of anger, see Kelly *et al.*, 2016).

The detrimental effect of left cerebellar TMS over participant’s ability to discriminate negative body emotional expressions was independent of body orientation (facing the observer *vs* averted orientation). Whereas Soria Bauser *et al.* (2012) have shown that electrophysiological correlates vary for averted and facing emotional bodies in the cerebrum, our data seem to suggest that cerebellar responses to body emotional postures may not be critically modulated by orientation of the observed agent. However, it is possible that orientation sensitivity in brain regions involved in action preparation depends on the specific response required (such as grasping *vs* simply pointing to an object), for which stimulus orientation may be more or less salient (Gutteling *et al.*, 2013). To shed light on body-orientation sensitivity in the cerebellum, it would be worth to combine cerebellar TMS with adaptation or priming paradigms, as it has been done in previous studies assessing orientation invariance in face-sensitive and object-sensitive regions in the cerebrum (Pitcher *et al.*, 2008; Silvanto *et al.*, 2010).

In Experiment 2 we reported a significant effect of orientation that was unrelated to the TMS itself, but was due to participants being better in discriminating between anger and sadness when bodies were presented averted by 45 degrees, compared to when they were directly facing the observer. This finding may appear at odd with prior literature suggesting that emotions are generally better recognized for faces (Guo and Shaw, 2015; Soria Bauser *et al.*, 2012) and bodies (Soria Bauser *et al.*, 2012) facing the observer. However, note that it should be noted that we selected facing and averted bodies from an available database (Thoma *et al.*, 2013) matching a priori (for each emotion) emotion recognizability between the averted and the facing orientation. We selected the stimuli in this way because we were not interested in possible effects of body orientation on emotion discrimination accuracy *per se* (these effects have already been investigated in prior studies, Soria Bauser *et al.*, 2012). In turn, we were interested in possible differences in cerebellar TMS effects depending on body perspective prompting the impression to be involved in a dyadic interpersonal interaction. In light of this, the better recognition of emotions expressed by averted *vs* facing bodies we reported in Experiment 2 is likely to be the spurious effect of (suboptimal) stimuli pre-selection.

Overall, participants in Experiment 2 were less accurate in discriminating between the positive emotions of surprise and happiness than between the negative emotions of sadness and anger (see Figure 3). One may thus wonder whether the lack of left cerebellar TMS effect for discrimination of the two positive emotions depended on accuracy being already low in this condition (although clearly above chance level) compared to the negative emotion condition. This explanation is unlikely. Indeed, previous studies showed that cerebellar involvement in cognitive tasks increases with the complexity and uncertainty of the task (Boecker *et al.*, 2002; D'Agata *et al.*, 2011; Schubotz and von Cramon, 2002; Volz *et al.*, 2003). Moreover, the impairment in emotion discrimination we observed across the two experiments is unlikely to have depended on unspecific effects of cerebellar stimulation over motor responses, or on generic interference on eye-movements control exerted by the cerebellum (e.g. Lynch and Tian, 2006; Ramat *et al.*, 2007). Indeed, if that were the case, TMS should have affected positive emotions discrimination (in Experiment 2) as well. Moreover, if cerebellar effects depended on indirect stimulation of the early visual cortex (see Renzi *et al.*, 2014), we should have observed a decrease in performance for early visual cortex stimulation as well, which was not the case (note that stimulation preceded the onset of the target stimulus so that no direct interference of early visual cortex stimulation was expected on the task). Finally, it should be noted that the TMS in our study affected accuracy but not response latencies. Selective effects of online TMS on accuracy or RTs are commonly reported in the literature (e.g. Cattaneo *et al.*, 2017; Ferrari *et al.*, 2018c; Pitcher *et al.*, 2009; Pitcher *et al.*, 2008) and may depend on the specific task at play (see Devlin and Watkins, 2007 for a discussion on differential effects of TMS over different behavioral indexes).

In summary, our findings suggest that the left cerebellum (Crus I/II) is involved in processing the negative emotional content conveyed by body postures and gestures. Future TMS studies may clarify the causal role of the cerebellum in processing different emotions beyond anger, and consider important emotional dimensions other than valence, such as arousal (e.g. Styliadis *et al.*, 2015), goal-directed tendencies (such as approach/avoidance) or emotions that are expressed by vocalizations and prosody (patients with cerebellar lesions may indeed show deficits in emotional prosody discrimination, see Adamaszek *et al.*, 2014). These studies will be of interest also in a clinical perspective, where non-invasive cerebellar stimulation is a promising tool in the treatment of motor, cognitive and affective deficits (van Dun and Manto, 2018).
